# Clinical Lipid Control Success Rate Before and After Percutaneous Coronary Intervention in Iran; a Single Center Study

**DOI:** 10.5812/ircmj.3370

**Published:** 2013-06-05

**Authors:** Seyed Kianoosh Hosseini, Maryam Tahvildari, Mohammad Javad Alemzadeh Ansari, Manouchehr Nakhjavani, Alireza Esteghamati, Masoumeh Lotfi Tokaldany

**Affiliations:** 1 Department of Cardiology, Tehran Heart Center, Tehran University of Medical Sciences, Tehran, IR Iran.; 2 Tehran University of Medical Sciences, Tehran, IR Iran.; 3 Endocrinology and Metabolism Research Centre (EMRC), Vali-Asr Hospital, Tehran University of Medical Sciences, Tehran, IR Iran.; 4 Research Department, Tehran Heart Center, Tehran University of Medical Sciences, Tehran, IR Iran

**Keywords:** Dyslipidemia, Lipid Control, Percutaneous Coronary Intervention

## Abstract

**Background:**

High cholesterol levels have long been considered an independent risk factor for cardiovascular disease (CVD).

**Objective:**

Controlling risk factors such as dyslipidemia in patients with coronary artery disease is necessary. We aimed to evaluate the success rate of lipid control, during 9 months follow-up after percutaneous coronary intervention (PCI).

**Patients and Methods:**

A total of 195 patients (67.7% men, mean age = 57.8 ± 9.4 years) who underwent PCI in Tehran Heart Center were included. Serum lipid profiles were measured in all the patients before PCI and at 9-month follow-up. Dyslipidemia was defined as serum levels of LDL-C ≥ 100 or TG ≥ 150 or TC ≥ 200 or HDL-C ≤ 40 mg/dl in the men and ≤ 50 mg/dl or less in the women, or non-HDL-C ≥ 130 mg/dl with or without the consumption of lipid-lowering agents. During follow up, all patients were given atorvastatin 20-40 mg/day.

**Results:**

Overall, 26.2% had diabetes mellitus, 42.6% had hypertension, and 34.9% were smokers. Dyslipidemia was more common in the women. At 9-month follow-up, there was no significant changes in terms of the prevalence of high HDL-C or low TG in patients; however, a significant increase was seen in the prevalence low TC in patients (63.6% vs. 80.5%; p value < 0.001), LDL-C (47.2% vs. 65.6%; p value < 0.001), and non-HDL-C (40.0% vs. 63.1%; p value < 0.001).

**Conclusions:**

Although by current treatments, the prevalence of patients with low TC, LDL-C and non-HDL-C has significantly increased; dyslipidemia persisted in a considerable proportion of patients. These results necessitate further investigations into the relationship between high serum lipids and long-term outcome of patients after PCI as well as further evaluations of the dyslipidemia treatment strategies.

## 1. Background

High cholesterol levels have long been considered an independent risk factor for cardiovascular disease (CVD) (1). It has been demonstrated that high total cholesterol (TC), high triglyceride (TG), high low-density lipoprotein cholesterol (LDL-C), low high-density lipoprotein cholesterol (HDL-C) and high non-HDL-C levels are associated with a high incidence rate of CVD (1-5). Patients undergoing percutaneous coronary intervention (PCI) are amongst the individuals with highest risk for developing adverse cardiac events and higher morbidity and mortality. Therefore, meticulous follow-up sessions and patient education are said to be crucial in further reaching the target levels of blood lipids in this high-risk group of patients. In spite of the emphasis of guidelines on the tight control of the aforementioned modifiable risk factors, several surveys have shown that still a large number of patients have not achieved their optimal status (6-9). There are different reports in various studies about the rates of dyslipidemia control (6, 8-11). The aim of this study was to evaluate the lipid profile levels (LDL-C, HDL-C, non-HDL-C, TC, and TG) amongst patients before PCI and during follow-up to estimate our success rates in the control of dyslipidemia. Strength of our study is our Iran population sample, which allows comparison between reportedly encountered data of developed countries and a different ethnicity of a developing country.

## 2. Objectives

Controlling risk factors such as dyslipidemia in patients with coronary artery disease is necessary. We aimed to evaluate the success rate of lipid control, during 9-month follow-up after percutaneous coronary intervention (PCI).

## 3. Patients and Methods

This retrospective study was conducted on patients who were referred by cardiologists to Tehran Heart Center both from inside and outside of the center and underwent PCI between 2003 and 2007. All the medical records of the patients were derived from the Interventional Registry carried out at Tehran Heart Center (THCR-IC). The databank contains patient data collected by cardiologists and trained general practitioners, and the validity of all the data is checked by re abstracting 10% of the patients entries and by reentering 5% of the patients' records. During this period, 4,732 patients underwent PCI, but full data on the lipid profile including before and after PCI were available in only 195 patients. Demographic characteristics and laboratory data were gathered at the time of the administration. This research was approved by the institutional review board, overseeing the participation of human subjects in research at Tehran University of Medical Sciences. This study conformed to the principles outlined in the Declaration of Helsinki. Lipid-lowering agent, atorvastatin 20-40 mg per day was commenced at the time of admission, if not given before hospitalization, and they were continued after PCI and discharge from hospital. All the patients were invited to return to our follow-up clinic. Follow-up information was obtained through direct clinical visits of the patients for 9 months after the procedures or from the referring physicians and telephone interviews. Dyslipidemia was positive if at least one of these conditions existed: TC of 200 mg/dl or more, LDL-C of 100 mg/dl or more, TG of 150 mg/dl or more, HDL-C of 40 mg/dl or less in men and 50 mg/dl or less in women, and non-HDL-C of 130 mg/dl or more. Achieving success in lipid control was defined as reaching the target levels for each of the above-mentioned components of the lipid profile. The lipid control success rates were evaluated before PCI and at 9-month follow-up.

### 3.1. Statistical Methods

The data are presented as mean ± SD for the continuous variables and as frequencies (percentage) for the categorical variables. The statistical package for social sciences (SPSS) version 15 software was used for data analysis. The continuous variables were compared using the Student t-test and the categorical variables were compared using the Chi-Square or Fisher exact test. The significant differences between the groups were determined at level < 0.05.

## 4. Results

**Figure 1.**
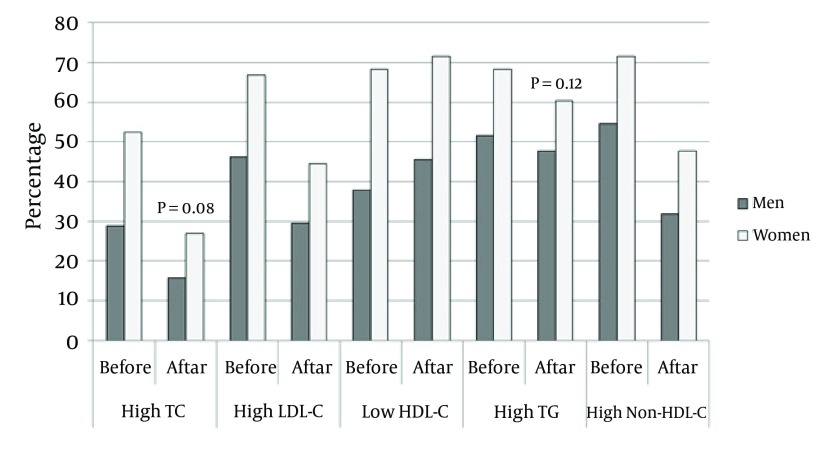
Comparison Between men and Women in Terms of the Prevalence of Dyslipidemia

Amongst the 195 patients, who underwent PCI, 132 (67.7%) were men. The baseline demographic characteristics of patients before PCI are listed in Table1. Except for smoking, all the other cardiovascular risk factors, including obesity, hypertension, diabetes mellitus, and dyslipidemia, were significantly more frequent in women than in men. In addition, the serum levels of TC, LDL-C, HDL-C and non-HDL-C were significantly higher in women. High TC, TG, LDL-C, non-HDL-C, and low HDL-C were seen in 36.4%, 56.9%, 52.8%, 60.0%, and 47.7% of the individuals, respectively. Figure 1 shows the prevalence of dyslipidemia before and after PCI. More women were dyslipidemic with respect to LDL-C, HDL-C, and non-HDL-C before and after PCI. The prevalence of patients with high TC and TG was higher in women than that in the men before PCI, but this difference disappeared after the follow-up (Figure 1). 

**Table 1.** Baseline Characteristics of 195 Patients Undergoing PCI

Characteristics	Total	Men (132, 67.7%)	Women (63, 32.3%)	P value
**Age, y, Mean ± SD**	57.8 ± 9.4	57.6 ± 9.8	58.4 ± 8.7	0.52
**BMI, Kg/m2, Mean ± SD**	27.5 ± 4.1	26.7 ± 3.4	29.4 ± 4.7	< 0.001
**Hypertension, No. (%)**	83 (42.6%)	43 (32.6%)	40 (63.5%)	< 0.001
**Diabetes Mellitus, No. (%)**	51 (26.2%)	23 (17.4%)	28 (44.4%)	< 0.001
**Smoking, No. (%)**	68 (34.9%)	64 (48.5%)	4 (6.4%)	< 0.001
**Family History for Premature CVD, No. (%)**	54 (27.7%)	33 (25%)	21 (33.3%)	0.22
**FBS, g/dl, Mean ± SD**	117.5 ± 48.7	111.0 ± 42.8	131.4 ± 57.4	0.008
**Hb, g/dl, Mean ± SD**	13.5 ± 1.7	14.1 ± 1.4	12.1 ± 1.4	< 0.001
**Lipid Profile, Mean ± SD**				
**TC**^**a**^**, mg/dl**	185.9 ± 49.5	179.2 ± 51.6	199.9 ± 41.8	0.006
**HDL-C**^**a**^**, mg/dl**	42.6 ± 9.4	41.4 ± 9.2	45.0 ± 9.6	0.01
**LDL-C**^**a**^**, mg/dl**	107.3 ± 42.3	102.3 ± 44.6	117.7 ± 35.2	0.01
**TG**^**a**^**, mg/dl**	175.5 ± 75.0	169.4 ± 72.2	188.3 ± 79.7	0.10
**Non-HDL-C**^**a**^**, mg/dl**	143.3 ± 46.9	137.8 ± 49.5	154.6 ± 38.8	0.01

^a^Abbreviations: TC, total cholesterol; HDL-C, high-density lipoprotein cholesterol; LDL-C, low-density lipoprotein cholesterol; TG, triglyceride; non-HDL-C, non high-density lipoprotein cholesterol

**Figure 2.**
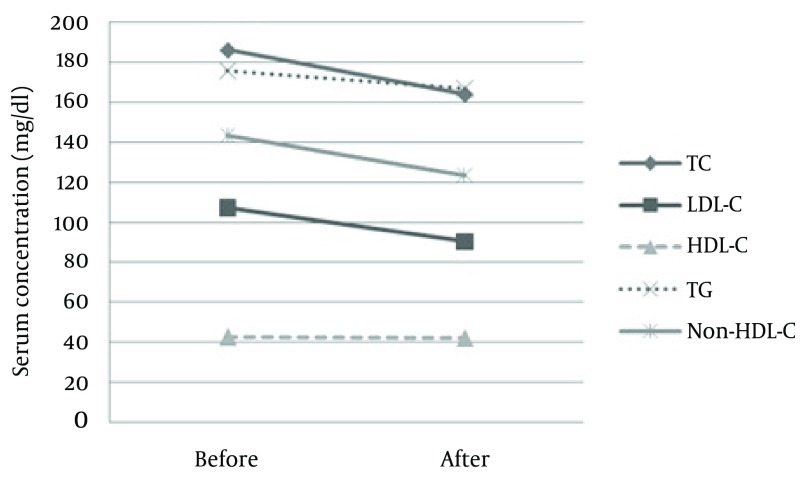
Serum Concentration of Lipids, Before and After PCI

Before and after PCI: More women dyslipidemic regarding LDL-C, HDL-C, and non-HDL-C before and after PCI. Although high TC and TG rates were more frequent in women than those in men before PCI, this difference disappeared after the follow-up period. After PCI and discharge from hospital, all the patients went through regular follow-up for 9 months. In this period, the serum concentration of TC, LDL-C and non-HDL-C decreased significantly (185.9 ± 49.5 mg/dl vs. 163.6 ± 40.9 mg/dl; P value < 0.001), (107.3 ± 42.3 mg/dl vs. 90.3 ± 41.4 mg/dl; P value < 0.001), and (143.3 ± 46.9 mg/dl vs. 123.5 ± 48.2 mg/dl; P value < 0.001) respectively, whereas there was no significant change in the concentration of HDL-C (42.6 ± 9.4 mg/dl vs. 42.0 ± 9.0; P value=0.48) and TG (175.5 ± 75.0 mg/dl vs. 166.7 ± 78.9; P value=0.12) (Figure 2). 

Although the serum concentration of TC, LDL-C and non-HDL-C decreased significantly after 9 months, there was no significant change in the concentration of HDL-C and TG. At the time of hospitalization, only 25 (12.8%) patients (22 men and 3 women; P value = 0.02) had all the measured serum lipids within the acceptable range, whilst after the follow-up period, this figure rose to 40 (20.5%) patients (32 men and 8 women; P value = 0.08). In contrast, 21 (10.8%) patients (7 men and 14 women; P value = 0.0006) had all the measured serum lipids within the uncontrolled range at the time of hospitalization, and 17 (8.7%) patients (8 men and 9 women; P value = 0.06) still remained dyslipidemic after the follow-up. Table 2 depicts the lipid control success rates before PCI and after the follow-up. A significant increase was observed in the prevalence of the patients who had low TC, LDL-C, and non-HDL-C after the follow-up, whereas no significant difference was found in the prevalence of the patients who had high HDL-C and low TG. 

**Table 2.** Lipid Control Success Rates before PCI and after Follow-up in 195 Patients (132 men and 63 Women)

Lipid Profile	Values	Sex	Before PCI, No. (%)	After PCI, No. (%)	P value
**TC**^**a**^	< 200 mg/dl	Men	94 (71.2%)	111 (84.1%)	0.01
		Women	30 (47.6%)	46 (73.0%)	0.006
		Total	124 (63.6%)	157 (80.5%)	< 0.001
**LDL-C**^**a**^	< 100 mg/dl	Men	71 (53.8%)	93 (70.5%)	0.007
		Women	21 (33.3%)	35 (55.6%)	0.01
		Total	92 (47.2%)	128 (65.6%)	< 0.001
**HDL-C**^**a**^	> 40 mg/dl in men, > 50 mg/dl in women	Men	82 (62.1%)	72 (54.5%)	0.26
		Women	20 (31.7%)	18 (28.6%)	0.84
		Total	102 (52.3%)	90 (46.2%)	0.27
**TG**^**a**^	< 150 mg/dl	Men	64 (48.5%)	69 (52.3%)	0.62
		Women	20 (31.7%)	25 (39.7%)	0.45
		Total	84 (43.1%)	94 (48.2%)	0.36
**Non-HDL-C**^**a**^	< 130 mg/dl	Men	60 (45.5%)	90 (68.2%)	< 0.001
		Women	18 (28.6%)	33 (52.4%)	0.01
		Total	78 (40.0%)	123 (63.1%)	< 0.001

^a^Abbreviations: TC, total cholesterol; HDL-C, high-density lipoprotein cholesterol; LDL-C, low-density lipoprotein cholesterol; TG, triglyceride; non-HDL-C, non high-density lipoprotein cholesterol

## 5. Discussion

Strong interdependence between high TC, high TG, high LDL-C, low HDL-C, and high non-HDL-C concentrations and CVD has been reported in previous studies (1-5, 12, 13). On the other hand, the prevalence of cardiovascular risk factors (particularly dyslipidemia) is high amongst the Iranian general population. Azizi et al. reported that the prevalence of high TC, low HDL-C and high TG, amongst the healthy Iranian population was 19.3%, 32%, and 5.3%, respectively (14). In 2008 Ghandehari et al. reported that the prevalence of high LDL-C, high TG, and low HDL-C amongst adult US persons was 28%, 13%, and 26% respectively (15). Ford et al. reported that the prevalence of high TG in the general population 20 years of age or older in the USA was 33.1% (16). Esteghamati et al. in 2006 showed that the prevalence of high TC, high LDL-C, low HDL-C, and high TG amongst Iranian patients with acute coronary syndrome was 41.8%, 63.8%, 74.4%, and 49%, respectively (17). Approximately a large proportion of our coronary artery disease patients, who underwent PCI, had high TC (36.4%), TG (56.9%), LDL-C (52.8%), and non-HDL-C (60.0%), as well as low HDL-C (47.7%), which chimes in with other studies (5, 17, 18). Pres et al. in 2010 stated that 60% of all their patients (diabetic and non-diabetic) with ST-elevation myocardial infarction treated with PCI had high TC; in addition, the prevalence of high TC in the diabetic patients with high LDL-C reached 78% and in the non-diabetic patients with high LDL-C amounted to 74%. The authors also reported that in their diabetic patients, in-hospital mortality was higher amongst those with elevated LDL-C levels and each increase in LDL-C on admission by 1 mmol/L (38.67 mg/dl) was related to an increase in in-hospital mortality (5). In our study, by current treatments and after 9 months of follow-up, a 16.9% increase was seen in the proportion of patients with low TC, 18.4% increase in the proportion of patients with low LDL-C, and 23.1% increase in the proportion of patients with low non-HDL-C, whereas there was a drop in the proportion of patients with high HDL-C and there was no significant increase in the proportion of patients with low TG after treatment. Khashayar et al. in 2010 showed that statin usage, albeit capable of reducing TC and LDL levels, did not exert an impact on TG and HDL levels, which is concordant with our results (19). Although after 9 months of follow-up, the proportion of patients with low TC, LDL-C, and non-HDL-C significantly increased in our study, despite using statins in all the patients, 19.5% had high TC, 34.4% high LDL-C, 53.8% low HDL-C, 51.8% high TG, and 36.9% high non-HDL-C. Furthermore, in a large proportion of our patients (79.5%), at least one of the components of the lipid profile remained uncontrollable; this is similar to or even more than that reported previously by other studies (6, 8-10). Stacy et al. in 2006 stated that approximately one third of their patients treated with lipid-lowering agents did not achieve individual lipid goals and two thirds of their patients did not attain the goal for all the three targets (LDL-C, HDL-C, and TG) (8). Goff et al., studying the prevalence, treatment and control of dyslipidemia in people free of clinical CVD, demonstrated that 29.3% of them had dyslipidemia and only 54.0% of them reported lipid-lowering drug therapy. Additionally, they showed that the control rate of dyslipidemia was achieved in 75.2% of the participants with treated dyslipidemia but in only 40.6% of all the participants with dyslipidemia (10). High prevalence of dyslipidemic patients after PCI in our study may be due to patients poor compliance or statin resistance (20). Amongst our 195 patients with established coronary artery disease, the cardiovascular risk factors, including high body mass index, hypertension, diabetes mellitus, and dyslipidemia, were more common in women; these findings were compatible with those reported by previous studies conducted in Iran (14, 17). There are varied concepts about gender-related dyslipidemia. For example, the Cruz et al. study in 2008 reported that the rate of dyslipidemia in patients with ischemic heart disease was equal in both men and women, (21) whereas the Goff et al. study showed that dyslipidemia in people free of known clinical CVD was more common in men than in women and that it was treated and controlled less often in men than in women (10). Our findings are compatible with those of the former study, which also stated that diabetic women had higher TC, LDL-C, TG, non-HDL-C, Lp (a), apo-B, and even HDL-C (22). Also in our study, the prevalence of women with high TC, LDL-C, TG, and non-HDL-C, as well as low HDL-C was significantly more than that in the men before PCI and after the follow-up; the only exceptions were high TC and TG after the follow-up period. The prevalence of men with low TC, LDL, and non-HDL-C increased significantly during the follow-up, which was the same as that in women. Despite treatment with atorvastatin, not only the proportion of the men or women with high HDL-C and low TG did not increase, but even a mild (non-significant) reduction (6.1%) was seen in the prevalence of patients with high HDL-C. Several large trials and meta-analyses have investigated the effects of lipid-lowering statin therapy and have consistently demonstrated that statins are highly effective for lowering LDL-C levels (11, 23-27). Despite achieving target LDL levels in patients treated with statins, they are in risk for the progression of atherosclerosis; this may be due to high TG and/or low HDL-C levels (28-30). In contrast, new findings suggest that although HDL-C levels are useful for initial cardiovascular risk assessment, they are not predictive of residual vascular risk amongst patients treated with potent statin therapy who attain very low LDL-C levels and in addition, there is no benefit from administrating niacin for this group of patients. Retrospective design and small sample size are two limitation of our study. In our study, at 9-month follow-up, the prevalence of patients with low TC, LDL-C and non-HDL-C significantly increased, whereas there was no significant increase in the prevalence of patients with high HDL-C or low TG. Lipid-lowering agents and regular follow-up visits did assist in achieving lipid-control goals in some patients, but a large proportion of the patients remained dyslipidemic at the end of the follow-up period. More attention should, therefore, be paid to life style modification, using statins with higher doses, and utilizing other classes of lipid-lowering agents such as ezetimibe or fibrates, if needed, in order to achieve the optimal targets of LDL-C, HDL-C, and TG. Further studies with prospective design and larger sample sizes dealing with role of lipid control in long term outcome of PCI is recommended.

**References**
